# A hybrid electro-optic polymer and TiO_2_ double-slot waveguide modulator

**DOI:** 10.1038/srep08561

**Published:** 2015-02-24

**Authors:** Feng Qiu, Andrew M. Spring, Daisuke Maeda, Masa-aki Ozawa, Keisuke Odoi, Akira Otomo, Isao Aoki, Shiyoshi Yokoyama

**Affiliations:** 1 Institute for Materials Chemistry and Engineering, Kyushu University, 6-1 Kasuga-koen Kasuga-city, Fukuoka, 816-8580 Japan; 2 Nissan Chemical Industries, LTD, 2-10-1 Tuboi Nishi, Funabashi, Chiba 274-8507, Japan; 3 National Institute of Information and Communications Technology, 588-2 Iwaoka, Nishi-ku, Kobe 651-2492, Japan

## Abstract

An electro-optic (EO) modulator using a TiO_2_ slot hybrid waveguide has been designed and fabricated. Optical mode calculations revealed that the mode was primarily confined within the slots when using a double-slot configuration, thus achieving a high EO activity experimentally. The TiO_2_ slots also acted as an important barrier to induce an enhanced DC field during the poling of the EO polymer and the driving of the EO modulator. The hybrid phase modulator exhibited a driving voltage (*V_π_*) of 1.6 V at 1550 nm, which can be further reduced to 0.8 V in a 1 cm-long push-pull Mach–Zehnder interferometer (MZI) structure. The modulator demonstrated a low propagation loss of 5 dB/cm and a relatively high end-fire coupling efficiency.

Organic EO polymers have been identified as one of the most promising class of materials for high-performance EO modulators due to their high EO coefficients (*r_33_*), ultrafast response times, low dielectric loss, and compatibility with other materials and substrates^1^,^2^,^3^,^4^. In particular, their excellent compatibility enables the construction of unique hybrid slot waveguides which are essential for the realization of integrated photonic devices. A typical slot waveguide consists of two narrow strip-lines of high refractive index material, which are separated by 100–300 nm from each other by a trench^5^. An efficient mode confinement in the slot is made possible by a large discontinuity of the optical field at the interface due to a high-low refractive index contrast, and a strong optical field within the polymer-filled slot^5^. Under these conditions, the optical interaction with the EO polymer is significantly enhanced. Another advantage can be seen in the electrode configuration. The optical mode is concentrated in the narrow slot space, while extending little to the outer edges. Such a mode confinement leads to a much smaller coplanar inter-electrode gap than that possible in a conventional EO polymer modulator^1^,^2^,^3^. Narrow electrodes enable a higher poling efficiency and a reduction of the driving voltage.

Considering these advantages, hybrid EO polymer slot modulators may promise a large figure of merit, such as a high poling efficiency, low driving voltage, low optical loss, and large bandwidth. Silicon-organic hybrid (SOH) waveguides have been sought in this field for several years due to the widespread utilization of CMOS silicon photonic wafers. Various types of SOH devices have been suggested and explored, including straight waveguide phase modulators^6^, MZI intensity modulators^7^,^8^, micro-ring modulators^9^, and photonic crystal hybrid modulators^10^,^11^,^12^. Considering the large refractive index contrast between silicon and EO polymers, approximately 30% of the optical mode is concentrated within the polymer-filled slot^7^,^11^,^12^. To drive the modulator, silicon strip-lines can be used themselves as the narrow-gap electrodes to apply the voltage to the EO polymer, therefore a few volts are enough to induce a π phase shift. So far, such modulators exhibited half-wave voltages (*V_π_*) of 0.25 V in a MZI and 0.97 V in a photonic crystal waveguide modulator^7^,^11^. Furthermore, an operation speed of several tens of GHz has been already reported^6^,^10^.

Despite this progress, some properties should be further considered for the widespread utilization of the SOH modulators. These include a propagation loss of about 20–35 dB/cm^6^,^9^, a relatively low in-device poling efficiency (30–50%) when using silicon strip-lines as electrodes^6^,^7^,^8^, and the requirement of an elaborate photonic structure such as a photonic crystal for the EO magnification^10^. The difficulty of in-device poling can be attributed to the leaking of current in the very narrow electrodes and the charge injection problem between silicon and the EO polymer interface. Therefore the necessity of improving poling efficiency in the narrow slot is suggested in the previous SOH report^6^,^7^. To improve the in-device *r*_33_, wide-slot photonic crystals have been explored^10^,^11^,^12^. The in-device EO activity is, therefore, capable of further improvement to reach EO activities greater than 100 pm/V^10^,^11^,^12^. The problem of high insertion loss is another barrier to realize the hybrid EO polymer modulator for interconnections. Direct coupling from silica fiber to the SOH waveguide facet is difficult without the use of special couplers. Both inverse tapered couplers and gratings have been proposed to improve the coupling efficiency^6^,^7^,^8^,^9^,^10^,^11^,^12^.

In order to overcome these obstacles there have been innovations targeted at waveguide design, fabrication, and materials development. Recently alternative high refractive index materials have been incorporated into photonic devices^13^,^14^,^15^. Titanium dioxide (TiO_2_), the material of choice in this study, has been shown to demonstrate a high transparency at the telecommunications band, good nonlinear optical activity, and can be used to fabricate a nanowire waveguide^14^,^15^,^16^. Its good transparency in the amorphous form ensures a low propagation loss of <1 dB/cm at 1550 nm. High-quality deposition of the thin film can be performed by using a conventional sputtering technique under controlled gas pressure and low temperatures^17^. Such versatility allows for the integration of photonic structures on various substrates and the blending with organic and polymer materials. The refractive index of TiO_2_ is lower than that of Si, but enough higher than that of EO polymer. Such a moderate index can be applied for the fabrication of a nanowire structure. Indeed, the loss improvement is suggested by using a thin TiO_2_ layer on the SOH device^18^. The electrical resistivity and dielectric constant are essential factors in the hybrid EO polymer. The electrical resistivity of TiO_2_ is several orders lower than that of the EO polymer, so that there is no electric barrier during the poling process^1^,^17^. Furthermore, the dielectric constant of TiO_2_ (>30) is higher than that of the EO polymer (~2). Such a large contrast might induce the enhanced DC field in the slot waveguide modulator.

In this work, we have fabricated a hybrid EO polymer and TiO_2_ slot waveguide, and investigated its prospective properties for the modulator application. The dimensions of the TiO_2_ slot were designed to enable the best optical mode confinement by using the beam propagation method (BPM) calculation. In addition, the distribution of the DC field across the slot is characterized by using the electromagnetic field calculation. Both DC field and optical mode calculations enable quantitative analysis of the overlap integral numerically. By the use of these calculations we successfully fabricated a double-slot modulator with the optimal EO properties. We measured a phase modulation with a *V*_π_ of 1.6 V at 1550 nm for the 1.5 cm-long electrode, which corresponds to an in-device *r*_33_ of 140 pm/V. The driving voltage can be converted to *V*_π_ = 1.1 V for the TE mode, and therefore reduced by half in the MZI application. The insertion loss of the fabricated double-slot modulator was 25 dB and the waveguide propagation loss was 5 dB/cm. Since the effective refractive index of the EO polymer/TiO_2_ slot is ~1.69, direct coupling of the light from a silica fiber to the waveguide facet is possible without any special coupler. This is a significant practical improvement towards a direct fiber-to-device connection, while the loss can be further reduced by the attachment of taper couplers.

## Results

### Modulator design and simulation

**Figure 1:**
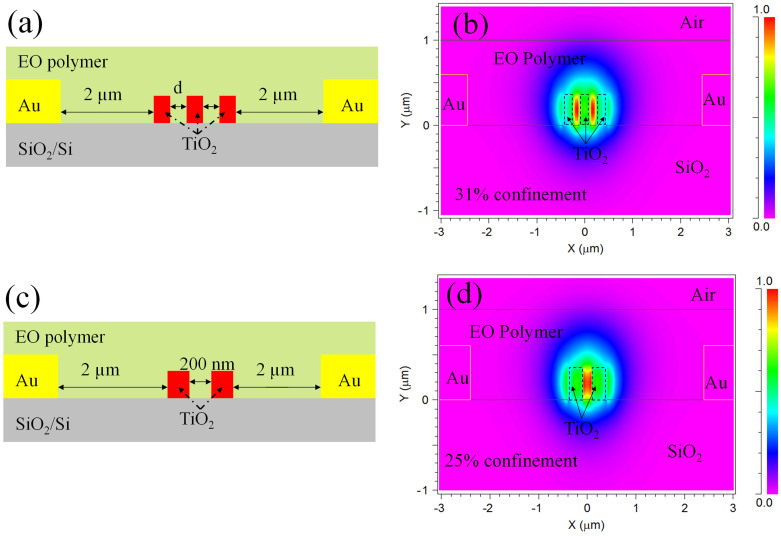
(a) Schematic and (b) TE_0_ mode distribution of the EO polymer filled TiO_2_ double-slot modulator when *d* = 150 nm; (c) single-slot structure and (d) its TE_0_ mode distribution are shown for comparison.

Figure 1(a) shows a schematic of the designed hybrid EO polymer and TiO_2_ double-slot modulator. The high refractive index ridges are constituted of three strip-lines each with a cross-section of 350 (height) × 200 (width) nm^2^. The distance from the TiO_2_ line to the electrode is 2.0 μm. The thicknesses of the EO polymer and Au electrode are 1.0 μm and 0.6 μm, respectively. Based on this schematic the TE_0_ mode distribution can be calculated using 3D BPM in Rsoft. In order to evaluate the best condition for the mode confinement, calculations were made for the slots by changing their width (*d*) between 100 and 500 nm. Figure 1(b) shows the mode confinement in the slots by setting *d* as 150 nm. In calculation, we used the measured refractive indices of 2.3 and 1.6 for TiO_2_ and EO polymer at 1550 nm, respectively. The calculation indicates that the cross-section of the optical mode is 2.0 μm and 1.0 μm in horizontal and vertical directions, respectively. The simulated mode size is much larger than the cross-section of the TiO_2_ strip-lines, whereas a large part of the optical mode extends into the EO polymer cladding (51%). In particular, it can be seen that an intense optical field is concentrated within the slots. The mode confinement was calculated as 31% in the slots, which exploits the increased optical interaction between the optical mode and the EO polymer. Combined with an effective refractive index of 1.69 in the EO polymer/TiO_2_ slot, end-fire coupling using a lensed silica fiber is possible with a sufficient coupling efficiency.

Here, it is worth to point out the utility of this double-slot modulator over the common slot structure. For comparison, the TiO_2_ single-slot is given in Fig. 1(c), and its TE_0_ mode distribution is shown in Fig. 1(d). The dimension of the TiO_2_ line is similar to that used in the double-slot, while *d* is set slightly larger for the optimization. The calculation indicates that the confinement factor of the optical mode in the slot is 25%, which is 6% smaller than that observed in the double-slot case. Calculation suggests that the double-slots offer a higher light interaction with the EO polymer than that in the single-slot structure.

The electrode geometry is another important parameter to induce the noncentrosymmetric orientation of the chromophore by applying the DC electric field, and to realize the modulator with a high in-device *r*_33_. The optical mode is parallel to the TE polarization, so the coplanar electrodes are placed across the slots with the certain distance. Since the electrical resistivity of the TiO_2_ (10^5^ Ωm) is several orders lower than that of the EO polymer (10^6−7^ Ωm), a voltage applied on the electrode can almost entirely drop to the EO polymer. Therefore, the narrow-gap electrodes are beneficial to perform effective poling and to achieve a low-voltage modulator driving, however too narrow electrodes may contribute to the optical loss due to Au absorption. In order to obtain a modulator with the best performance, i.e. low *V*_π_ and low loss, the optical mode should be concentrated around the core as much as possible, as the result the inter-electrode gap can be set for the minimum.

**Figure 2:**
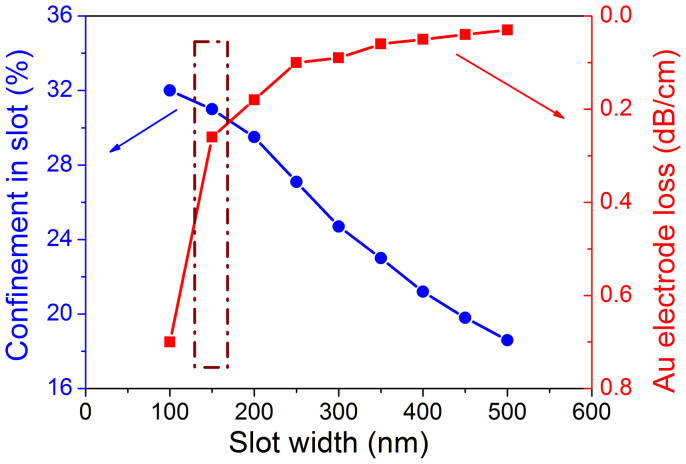
Calculated electrode absorption loss and light confinement versus slot width.

In order to clarify the double-slots geometry and its suitable inter-electrode gap, the confinement factors of the optical mode are calculated for the slots with different *d*. The optical losses are also obtained under the same slots' condition, in which the slot-electrode distance is set as 2.0 μm. In Fig. 2, there is a clear trade-off between the confinement factor and the optical loss between *d* = 100 and 500 nm. The highest confinement factor of 33% is obtained for *d* = 100 nm. In this case, however, the optical loss is rather large (0.7 dB/cm). The optical loss steeply diminishes as the slot-gap is slightly increased, so that 0.25 dB/cm is obtained for *d* = 150 nm, while the confinement factor remains high (31%). For *d* > 250 nm, the change in the optical loss reaches a plateau and finally decreases to the smallest level for *d* = 500 nm, the confinement factor decreases linearly in wider slots. Therefore, we chose *d* = 150 nm for the fabrication, expecting a small optical loss and a high enough confinement of the optical mode in the double-slots.

**Figure 3:**
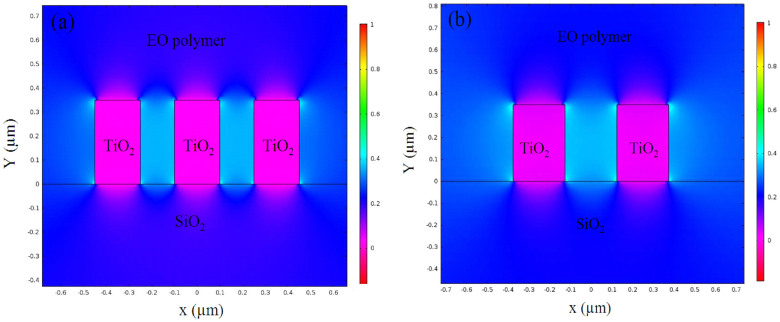
Simulated DC electrical filed distributions around the slots of the (a) double-slot and (b) single-slot modulator.

In the hybrid EO polymer and TiO_2_ slot waveguide, discontinuous electric field distributions can be expected at the boundary between the TiO_2_ and the EO polymer. Therefore, we clarified the DC field across the electrodes and considered the achievable *r*_33_ as a result of the poling process. The DC field in the double-slots modulator was simulated using COMSOL 4.4, and its relative field intensity across the slots is shown in Fig. 3(a). In the calculation, the reported dielectric constants of 3.0 and 60 are used for the EO polymer and the TiO_2_, respectively^3^,^19^. For comparison, the calculation for a single-slot modulator is given in Fig. 3(b). In both slot structures, the high-contrast field discontinuity can be seen at the boundary between the TiO_2_ and the EO polymer. Beside the TiO_2_ lines, a relatively high DC field appears in the EO polymer. In particular, the slots cause a significant increase in the DC field intensity, in which ~35% enhancement can be estimated relative to other EO polymer regions. By comparing Fig. 3 with Fig. 1(b) and (d), one can see that the DC and optical fields in the slots overlap well. Such an emphasis of the DC field is beneficial to both the poling process and the low-voltage driving of the modulator.

### Modulator Test

**Figure 4:**
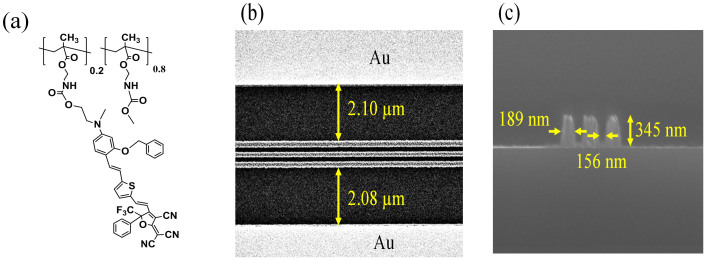
(a) Molecular structure of the EO polymer, (b) top-view and (c) cross-section of the slot waveguide taken by SEM.

Figure 4 (b) and (c) shows the top view and a cross-section of the TiO_2_ double-slots waveguide taken by SEM. The EO polymer, structure shown in Fig. 4 (a), was spin-coated on the waveguide to form a 1.0 μm-thick film. To induce an EO effect, the EO polymer in the modulator was poled at 140°C by applying an electric field of 80 V/μm to the electrodes. The fiber-to fiber insertion loss at 1550 nm for the TE polarization was 25 dB as the sum of the propagation loss and the coupling loss. The propagation loss was measured as ~5 dB/cm by using the cut-back method. We found little change in the loss before and after poling. The propagation loss mainly originates from the EO polymer absorption (3 dB/cm) and the scattering at etched TiO_2_ sidewalls which may be reduced by improving the etching process. Since the spot diameter (4 μm) from the lensed fiber is a little larger than the calculated mode size (2.0 μm × 1.0 μm), the insertion loss can be reduced by the introduction of taper couplers.

**Figure 5:**
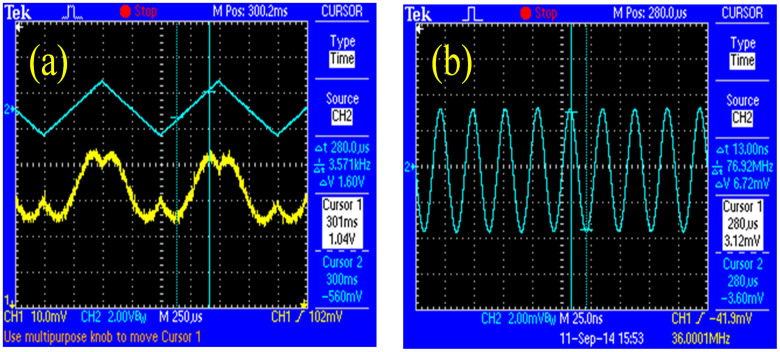
(a) Measured transfer function of the modulator at 1 kHz (Top: applied triangular voltage waveform. Bottom: optical output from the modulator). (b) Oscilloscope spectrum of 36 MHz signal output from the modulator.

The fabricated phase modulator was tested as an intensity modulator through a cross-polarization setup. The laser light at 1550 nm with a +45° linear polarization was coupled into the modulator through a polarization-maintaining lensed fiber. Output light from the modulator was passed a −45° polarizer, and then collected by a photo-detector. The light intensity was measured with an applied triangular voltage waveform at a frequency of 1 kHz as shown in Fig. 5(a). From a clear modulation output function of the modulator, a *V_π_* of 1.6 V was measured. Considering *r_33_* = 3*r_13_* in an EO polymer^20^,^21^, the true *V_π_* in the TE mode can be reduced to 1.1 V by a factor of 2/3. For the reference experiment, the TiO_2_ single-slot waveguide was fabricated and the modulator was prepared after poling the EO polymer in the same manner. However, the measured *V*_π_ in the TE mode was 5 V. This result shows clear evidence that the EO activity is significantly enhanced in the double-slot configuration than the single-slot. We can convert the TE mode double-slot waveguide modulator into the push-pull MZI modulator, which allows for a *V_π_* = 0.8 V in a 1 cm-long electrode configuration. Such a predicted figure-of-merit is at a competitive level with the state-of-the-art SOH photonic crystal slot modulators^7^,^11^. Here, it is worth noting that using the TiO_2_ double-slot EO modulator enables the operation with a large optical bandwidth, which is generally restricted in the photonic crystal devices due to their narrow stop band property^10^,^11^,^12^.

By using the measured *V_π_*, the electrode length, and the overlap factor of ~45% between the optical field and the applied electrical field, an effective in-device *r_33_* was estimated to be ~140 pm/V. Obtained in-device *r_33_* is 55% higher than the in-film *r_33_* measured by using the Teng-Man reflection method^20^. This overall improvement in the in-device *r_33_* can be attributed to two important functions of the TiO_2_ slots. The first is the DC field enhancement around the TiO_2_ slots as shown in Fig. 3(a). The second is the charge injection blocking effect by the TiO_2_ strip-lines, which limits the excess charge injection from the electrode into the EO polymer and reduces the current leakage^22^. The EO polymer can therefore be highly pooled to provide the greatest EO activity.

In order to evaluate the modulator response to the RF signal, a sinusoidal voltage was applied to one electrode and the other used as the ground. The output optical signal was measured by a photo-detector connected to an oscilloscope. Figure 5(b) is the obtained modulator response to 36 MHz by fine tuning the device with an applied bias voltage of 0.9 V. In this lumped modulator, the electrode structure is not specially designed and fabricated for the high-RF purpose. The signal response at higher speeds became imprecise due to a large signal intensity loss. Utilizing the traveling-wave-electrodes should enable a further increase in the operating speed of up to multi-GHz^23^.

In summary, we have successfully designed and demonstrated an EO polymer cladded TiO_2_ double-slot waveguide phase modulator. In the designed modulator, ~31% of the TE mode can be confined within the slots and all the poling voltage can be applied to the EO polymer. Because of the large dielectric contrast between the TiO_2_ and the EO polymer, an enhanced DC field can arise in the slots. In addition, the TiO_2_ can also block the excessive charge injection and reduce the current leakage. As a result, an effective poling of the EO polymer and a low *V_π_* of 1.6 V were realized. By utilizing a traveling-wave-electrode in the future, the modulator can be applied in broadband modulator applications. Based on the EO polymer filled TiO_2_ double-slot waveguide, other useful optical structures for EO modulators, such as ring, MZI, and photonic crystal, can also be readily realized.

## Methods

The hybrid EO polymer and TiO_2_ double-slot modulator was fabricated as follows. Firstly a 350 nm thick TiO_2_ section was deposited onto a SiO_2_ (2 μm)/Si (500 μm) substrate using RF sputtering of a TiO_2_ target under controlled Ar/O_2_ gas pressure. The temperature of the substrate was kept below 85°C during the deposition in order to obtain the amorphous form of the TiO_2_ film. The slot configurations were fabricated using electron-beam lithography and reactive ion etching with CHF_3_ gas. Subsequently, 1.5 cm-long gold electrodes were patterned by lift-off process as the designed coplanar structure. Finally, the EO polymer (structure in Fig. 4(a)) was spin-coated, and then baked at 120°C for 48 hours to form a 1 μm thick film. The EO polymer was prepared according to our previous methods^24^. The polymer has a chromophore loading density of approximately 40 wt%, a glass transition temperature of 135°C, and a thermal decomposition temperature of 296°C.

## Author Contributions

F.Q. conceived the original idea. F.Q. and S.Y. performed the simulations and measurement. A.S., D.M., M.O., K.O., A.O. and I.A. contributed to scientific discussions of the manuscript. All authors reviewed this manuscript.
